# Supplementary material for: Hybridization-encoded DNA tags with paper-based readout for anti-forgery raw material tracking
Source: Nat Commun. 2025 Jul 1;16:5832. doi: 10.1038/s41467-025-60282-7 (PMC12214956; doi:10.1038/s41467-025-60282-7)
Supplement: Supplementary file 1 — Supplementary Information [file 41467_2025_60282_MOESM1_ESM.pdf]

Supplementary Information: Hybridization-Encoded DNA Tags with Paper-Based Readout for Anti-Forgery Raw Material Tracking

Table S1. DNA sequences used in our experiments.

Oligo Name	Sequence
FAM_S0*	/56-FAM/TAGTGAGTTTGAATAAGAGGTT
U15_T	CTCTCCTTATCAAACCACCAC
S0_S1_U15_T	CCTCTTATTCAAACTCACTAACTCCATCATATTACATCACCTCTCCTTATCAAACC ACCAC
T30_U15*_S1*	TTTTTTTTTTTTTTTTTTTTTTTTTTTTTTGTTTGATAAGGAGAGGTGATGTAAT ATGATGGAGT
T*_U15*_S1*	GTGGTGGTTTGATAAGGAGAGGTGATGTAATATGATGGAGT
S0_S2_U15_T	CCTCTTATTCAAACTCACTATCACTATAACTTATCCACACCTCTCCTTATCAAACC ACCAC
T30_U15*_S2*	TTTTTTTTTTTTTTTTTTTTTTTTTTTTTTGTTTGATAAGGAGAGGTGTGGATAA GTTATAGTGA
T*_U15*_S2*	GTGGTGGTTTGATAAGGAGAGGTGTGGATAAGTTATAGTGA
S0_S3_U15_T	CCTCTTATTCAAACTCACTACTCACTAAACTTTAACCTCACTCTCCTTATCAAACC ACCAC
T30_U15*_S3*	TTTTTTTTTTTTTTTTTTTTTTTTTTTTTTGTTTGATAAGGAGAGTGAGGTTAAA GTTTAGTGAG
T*_U15*_S3*	GTGGTGGTTTGATAAGGAGAGTGAGGTTAAAGTTTAGTGAG
S0_S5_U15_T	CCTCTTATTCAAACTCACTAAACCTCTTCAAACCTTCCTACTCTCCTTATCAAAC CACCAC
T30_U15*_S5*	TTTTTTTTTTTTTTTTTTTTTTTTTTTTTTGTTTGATAAGGAGAGTAGGAAGGT TTGAAGAGGTT
T*_U15*_S5*	GTGGTGGTTTGATAAGGAGAGTAGGAAGGTTTGAAGAGGTT
S0_S6_U15_T	CCTCTTATTCAAACTCACTAACATCCTCCACATCACTCACCTCTCCTTATCAAACC ACCAC
T30_U15*_S6*	TTTTTTTTTTTTTTTTTTTTTTTTTTTTTTGTTTGATAAGGAGAGGTGAGTGATG TGGAGGATGT
T*_U15*_S6*	GTGGTGGTTTGATAAGGAGAGGTGAGTGATGTGGAGGATGT
S0_S7_U15_T	CCTCTTATTCAAACTCACTATTCTACTTAACACTCTCCATCTCTCCTTATCAAACC ACCAC
T30_U15*_S7*	TTTTTTTTTTTTTTTTTTTTTTTTTTTTTTGTTTGATAAGGAGAGATGGAGAGTG TTAAGTAGAA
T*_U15*_S7*	GTGGTGGTTTGATAAGGAGAGATGGAGAGTGTTAAGTAGAA
S0_S8_U15_T	CCTCTTATTCAAACTCACTACCTTCACTCCAACAATATTCCTCTCCTTATCAAACC ACCAC
T30_U15*_S8*	TTTTTTTTTTTTTTTTTTTTTTTTTTTTTTGTTTGATAAGGAGAGGAATATTGTT GGAGTGAAGG
T*_U15*_S8*	GTGGTGGTTTGATAAGGAGAGGAATATTGTTGGAGTGAAGG
S0_S9_U15_T	CCTCTTATTCAAACTCACTATCCTTAACTCATCCTCCTCTCTCCTTATCAAACC ACCAC
T30_U15*_S9*	TTTTTTTTTTTTTTTTTTTTTTTTTTTTTTGTTTGATAAGGAGAGAGAGGAGGAT GAGTTAAGGA
T*_U15*_S9*	GTGGTGGTTTGATAAGGAGAGAGAGGAGGATGAGTTAAGGA
S0_S10_U15_T	CCTCTTATTCAAACTCACTATACTATCATTTCCACCACCACTCTCCTTATCAAACC ACCAC
T30_U15*_S10*	TTTTTTTTTTTTTTTTTTTTTTTTTTTTTTGTTTGATAAGGAGAGTGGTGGTGGA AATGATAGTA
T*_U15*_S10*	GTGGTGGTTTGATAAGGAGAGTGGTGGTGGAAATGATAGTA
S0_S12_U15_T	CCTCTTATTCAAACTCACTAACACCAATTCCTCTACTTCCCTCTCCTTATCAAACC ACCAC

T30_U15*_S12*	TTTTTTTTTTTTTTTTTTTTTTTTTTTTTTGTTTGATAAGGAGAGGGAAGTAGAG GAATTGGTGT
T*_U15*_S12*	GTGGTGGTTTGATAAGGAGAGGGAAGTAGAGGAATTGGTGT
S0_S13_U15_T	CCTCTTATTCAAACTCACTAAACTCTTCTCCATACCAACACTCTCCTTATCAAACC ACCAC
T30_U15*_S13*	TTTTTTTTTTTTTTTTTTTTTTTTTTTTTTGTTTGATAAGGAGAGTGTTGGTATG GAGAAGAGTT
T*_U15*_S13*	GTGGTGGTTTGATAAGGAGAGTGTTGGTATGGAGAAGAGTT
S0_S14_U15_T	CCTCTTATTCAAACTCACTATTCCTCTTCTTCTCCAATTTCTCTCCTTATCAAACC ACCAC
T30_U15*_S14*	TTTTTTTTTTTTTTTTTTTTTTTTTTTTTTGTTTGATAAGGAGAGAAATTGGAGA AGAAGAGGAA
T*_U15*_S14*	GTGGTGGTTTGATAAGGAGAGAAATTGGAGAAGAAGAGGAA
S0_S15_U15_T	CCTCTTATTCAAACTCACTAAAACTATACACCTCACTCTACTCTCCTTATCAAACC ACCAC
T30_U15*_S15*	TTTTTTTTTTTTTTTTTTTTTTTTTTTTTTGTTTGATAAGGAGAGTAGAGTGAGG TGTATAGTTT
T*_U15*_S15*	GTGGTGGTTTGATAAGGAGAGTAGAGTGAGGTGTATAGTTT
S0_S16_U15_T	CCTCTTATTCAAACTCACTAACCTCATACTCACCATACATCTCTCCTTATCAAACC ACCAC
T30_U15*_S16*	TTTTTTTTTTTTTTTTTTTTTTTTTTTTTTGTTTGATAAGGAGAGATGTATGGTG AGTATGAGGT
T*_U15*_S16*	GTGGTGGTTTGATAAGGAGAGATGTATGGTGAGTATGAGGT
S0_S17_U15_T	CCTCTTATTCAAACTCACTATTCACTCACTATCACACTTCCTCTCCTTATCAAACC ACCAC
T30_U15*_S17*	TTTTTTTTTTTTTTTTTTTTTTTTTTTTTTGTTTGATAAGGAGAGGAAGTGTGAT AGTGAGTGAA
T*_U15*_S17*	GTGGTGGTTTGATAAGGAGAGGAAGTGTGATAGTGAGTGAA
S0_S18_U15_T	CCTCTTATTCAAACTCACTACACCAAATCAACTCCTCTCTCTCTCCTTATCAAACC ACCAC
T30_U15*_S18*	TTTTTTTTTTTTTTTTTTTTTTTTTTTTTTGTTTGATAAGGAGAGAGAGAGGAGT TGATTTGGTG
T*_U15*_S18*	GTGGTGGTTTGATAAGGAGAGAGAGAGGAGTTGATTTGGTG
S0_S19_U15_T	CCTCTTATTCAAACTCACTAACCAATATACTCCAAACTCCCTCTCCTTATCAAACC ACCAC
T30_U15*_S19*	TTTTTTTTTTTTTTTTTTTTTTTTTTTTTTGTTTGATAAGGAGAGGGAGTTTGGA GTATATTGGT
T*_U15*_S19*	GTGGTGGTTTGATAAGGAGAGGGAGTTTGGAGTATATTGGT
S0_S20_U15_T	CCTCTTATTCAAACTCACTACAACCTCCTTCCAATCCTATCTCTCCTTATCAAACC ACCAC
T30_U15*_S20*	TTTTTTTTTTTTTTTTTTTTTTTTTTTTTTGTTTGATAAGGAGAGATAGGATTGG AAGGAGGTTG
T*_U15*_S20*	GTGGTGGTTTGATAAGGAGAGATAGGATTGGAAGGAGGTTG
S0_S21_U15_T	CCTCTTATTCAAACTCACTACTCCTACCATCACCTCATATCTCTCCTTATCAAACC ACCAC
T30_U15*_S21*	TTTTTTTTTTTTTTTTTTTTTTTTTTTTTTGTTTGATAAGGAGAGATATGAGGTG ATGGTAGGAG
T*_U15*_S21*	GTGGTGGTTTGATAAGGAGAGATATGAGGTGATGGTAGGAG
S0_S22_U15_T	CCTCTTATTCAAACTCACTAACACCTACACTATTCAACAACTCTCCTTATCAAACC ACCAC
T30_U15*_S22*	TTTTTTTTTTTTTTTTTTTTTTTTTTTTTTGTTTGATAAGGAGAGTTGTTGAATA GTGTAGGTGT

T*_U15*_S22*	GTGGTGGTTTGATAAGGAGAGTTGTTGAATAGTGTAGGTGT
S0_S23_U15_T	CCTCTTATTCAAACTCACTATACCTCCTACCAAACCAAACCTCTCCTTATCAAACCACCAC
T30_U15*_S23*	TTTTTTTTTTTTTTTTTTTTTTTTTTTTTTGTTTGATAAGGAGAGGTTTGGTTTGGTAGGAGGTA
T*_U15*_S23*	GTGGTGGTTTGATAAGGAGAGGTTTGGTTTGGTAGGAGGTA
S0_S27_U15_T	CCTCTTATTCAAACTCACTACCAACACTATCTTCCAAACCCTCTCCTTATCAAACCACCAC
T30_U15*_S27*	TTTTTTTTTTTTTTTTTTTTTTTTTTTTTTGTTTGATAAGGAGAGGGTTTGGAAGATAGTGTTGG
T*_U15*_S27*	GTGGTGGTTTGATAAGGAGAGGGTTTGGAAGATAGTGTTGG
S0_S29_U15_T	CCTCTTATTCAAACTCACTAACCACTCATCTATTTCAACACTCTCCTTATCAAACCACCAC
T30_U15*_S29*	TTTTTTTTTTTTTTTTTTTTTTTTTTTTTTGTTTGATAAGGAGAGTGTTGAAATAGATGAGTGGT
T*_U15*_S29*	GTGGTGGTTTGATAAGGAGAGTGTTGAAATAGATGAGTGGT
S0_S30_U15_T	CCTCTTATTCAAACTCACTATCTCTCTATACCTATTTACCCTCTCCTTATCAAACCACCAC
T30_U15*_S30*	TTTTTTTTTTTTTTTTTTTTTTTTTTTTTTGTTTGATAAGGAGAGGGTAAATAGGTATAGAGAGA
T*_U15*_S30*	GTGGTGGTTTGATAAGGAGAGGGTAAATAGGTATAGAGAGA
S0_S31_U15_T	CCTCTTATTCAAACTCACTACACCACACTACCACATACAACTCTCCTTATCAAACCACCAC
T30_U15*_S31*	TTTTTTTTTTTTTTTTTTTTTTTTTTTTTTGTTTGATAAGGAGAGTTGTATGTGGTAGTGTGGTG
T*_U15*_S31*	GTGGTGGTTTGATAAGGAGAGTTGTATGTGGTAGTGTGGTG
5phoRP*3AM	/5phos/GAGGTAAAGTTGTGATGAA/3AmMO/
FP	GTGGTGGTTTGATAAGG
U11_T_FP*	/5phos/CCTTATCAAACCACCAC

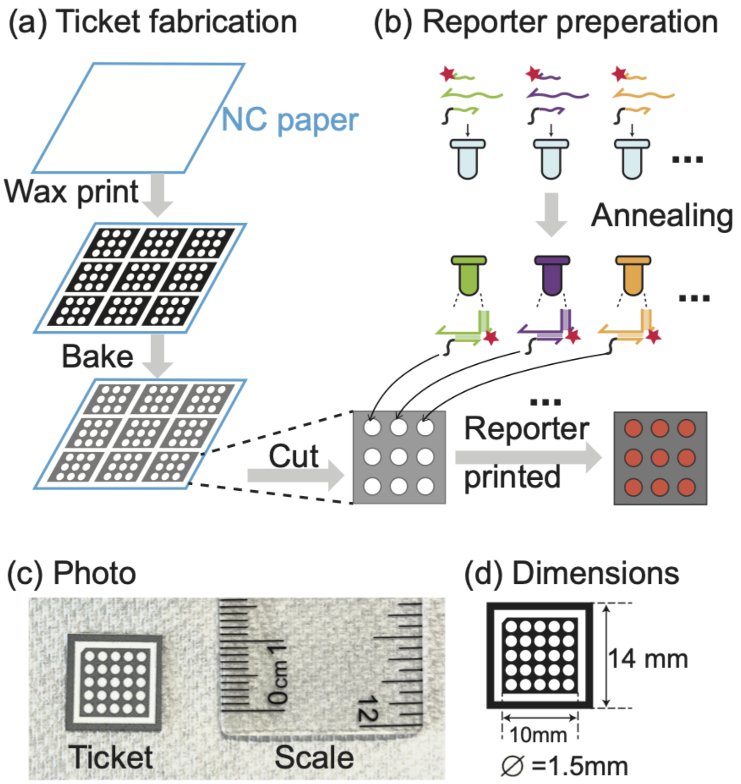

Figure S1: Fabrication of the DNATag reading ticket. (a) Ticket fabrication starts with printing designed ticket patterns onto the nitrocellulose (NC) paper using a wax printer. The printed paper was then baked at 90∘C for 10 minutes and cut into individual tickets. (b) The DNA strands that compose the reporter complexes were mixed in a tube and went through the annealing protocol for hybridization assembly. Next, the reporter complexes were pipetted onto the spots of the ticket and dried at room temperature for at least 20 minutes. The reporter ticket was then washed by flipping it over several times in water, dried again, and was ready for use. (c) Unretouched photo of the fabricated ticket, with a ruler next to it for size comparison. (d) Design and dimensions of the wax print. The wax is hydrophobic and worsens the contact between the ticket and the DNATag, so a surrounding blank groove was designed to act as a hydrophilic barrier that helps maintain the contact between the DNATag solution and the ticket.

	1	2	3	4	5
A	T1	T2	T3		
B		NC1	NC1		
C	T4	T5	T6	T7	
D		NC2	NC2		
E					

Name	DNABit Conc	Reporter Conc
T1	10 µM	5
T2	5 µM	5
T3	3 µM	5
T4	10 µM	2
T5	5 µM	2
T6	3 µM	2
T7	1 µM	2
NC1	0	5
NC2	0	2

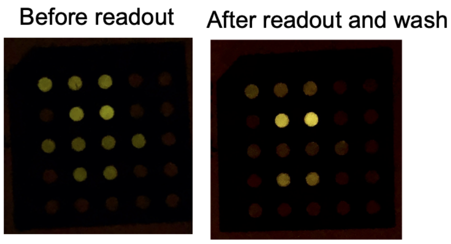

Figure S2: Sensitivity test with varied concentrations of the reporter complex and the DNAbits. According to the result, reporter concentration of 2 μM and DNAbit concentration of 3 μM are sufficient for a significant readout.

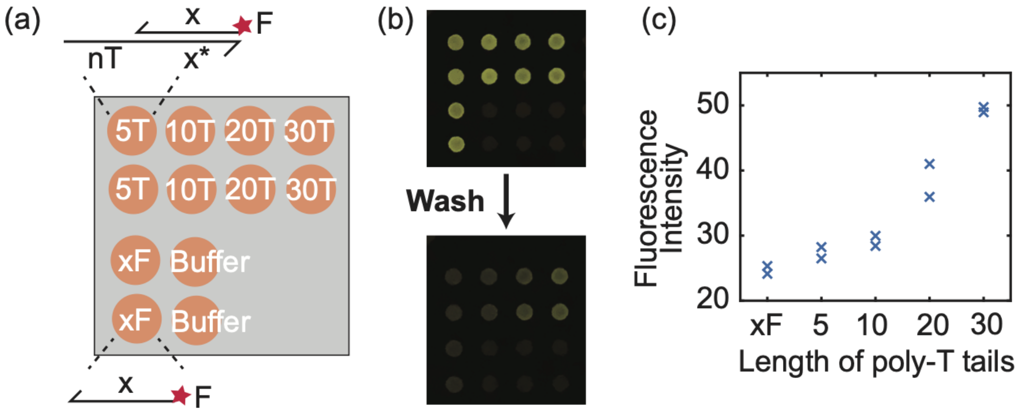

Figure S3: Testing binding affinity of DNA molecules with poly-T tails of different lengths to the nitrocellulose paper. (a) A poly-T tail of varied lengths (5, 10, 20, and 30 T nucleotides) was attached to oligo X^*, which hybridized to a fluorophore-labeled oligo X. The oligo dimmers with were pipetted onto spots of a NC ticket, each replicated for robustness. After all drops were completely dried, the ticket was washed in water and dried again before imaging. (b) Fluorescent images of the ticket before and after the wash. (c) The longer the poly-T tail, the more fluorescence signal remained on the paper after wash, suggesting a stronger binding affinity of the poly-T tails to the NC paper.

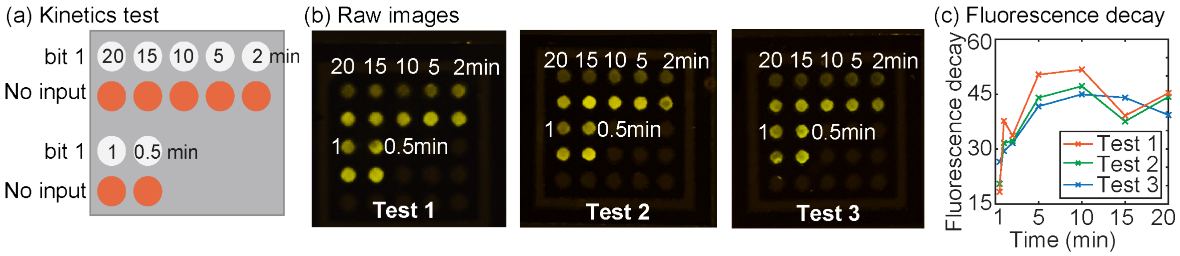

Figure S4: Triplicated kinetics test of the reaction between DNA Bit 1 and the reporter on the ticket. (a) Experimental layout. (b) Raw images of the DNATag reading tickets after the reaction. Each ticket was loaded with a reporter assembly carrying a unique Si domain and was applied its corresponding DNA Bit 1 or buffer as negative control. (c) Fluorescence decay plotted versus time in linear scale. The reactions all exceeded their half life within 5 minutes and saturated around 10 minutes.

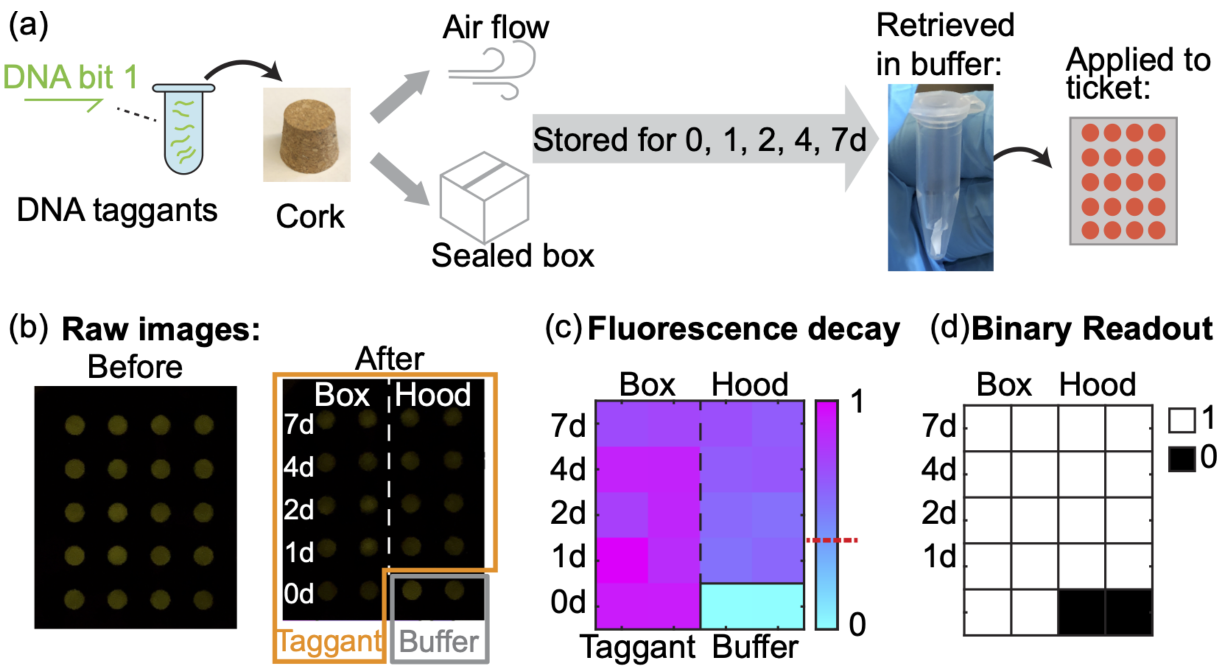

Figure S5: Stability test of DNA Bits on object surfaces. (a) DNA Bit 1s were left on the cork surfaces in the chemical hood or in a sealed box for 0, 1, 2, 4, and 7 days before rehydrated and applied to the reporter ticket. (b) Images of the reporter ticket before and after applying the DNA tags retrieved from cork surfaces. All groups were duplicated in columns for robustness. (c) Heatmap of the fluorescence decay. (d) Binary readout.

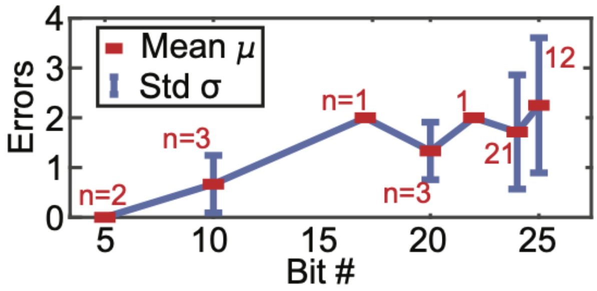

Figure S6: Raw error counts from the reading tickets from all multi-bit experiments we performed. The numbers of experiments performed with each bit # are labeled in red. Error bars represent standard deviation.

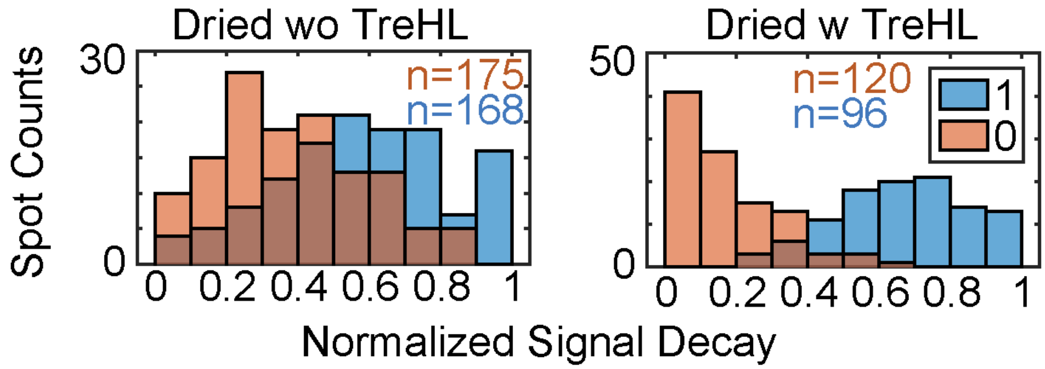

Figure S7: Comparison of the analog data reading DNATags that have been dried with (right) or without (left) trehalose.

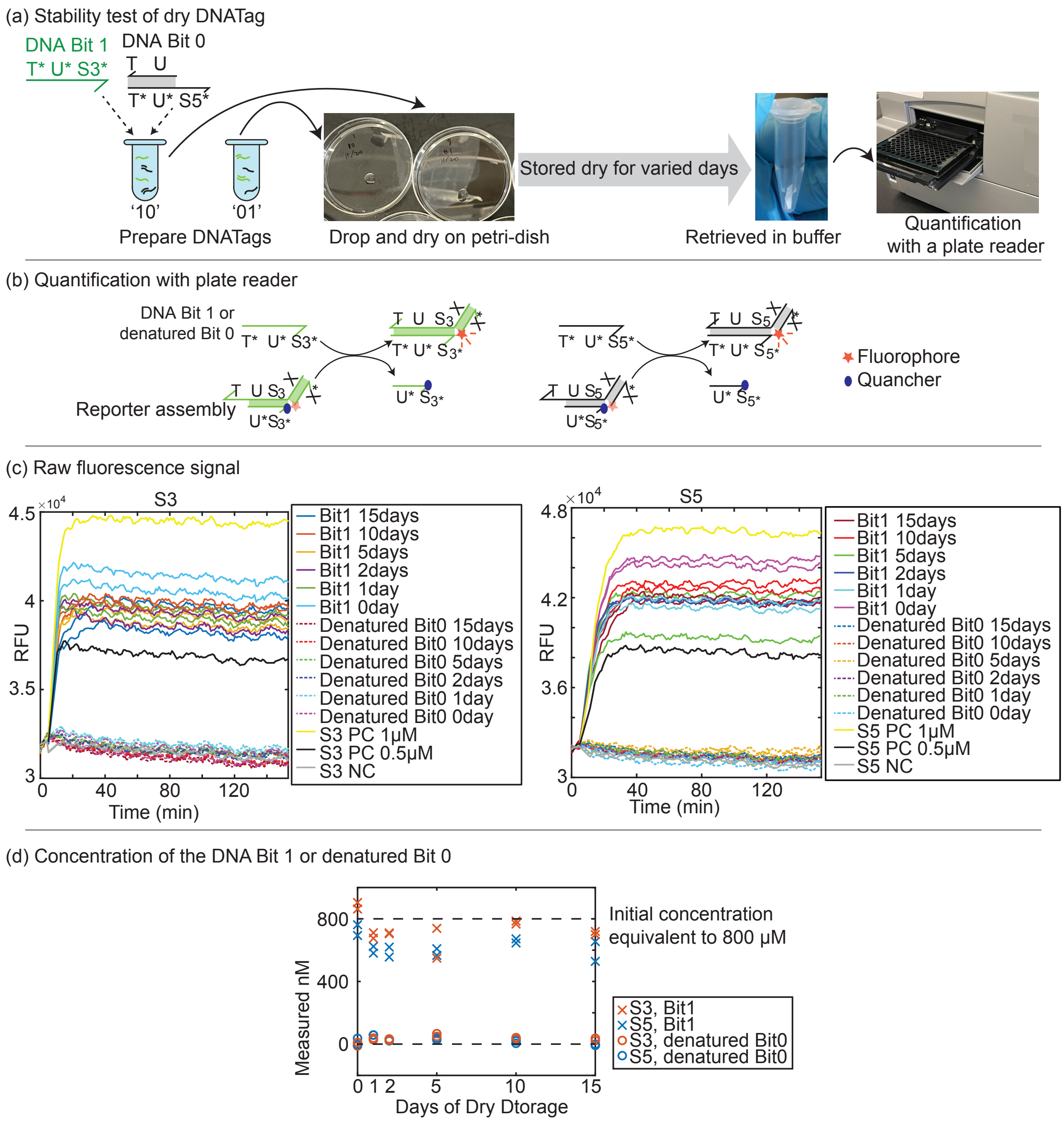

Figure S8: Stability test of dry DNATags. (a) Experimental workflow of the dry stability test. We prepared two 2-bit DNATag solutions '10' and '01' with DNA Bit strands carrying S3* and S5* domains, representing the two digits respectively. The DNATag solutions were freshly prepared and dried on a petri-dish at varied dates. After storage at dry state at room temperature, all DNATags were rehydrated and retrieved back in solution on the same day. The retrieved DNATags were then quantified using the corresponding fluorescent reporters in a microplate reader. Positive controls (pure DNA Bit 1 strand at 1μM and 0.5 μM) and negative controls (buffer without any DNA) were also presented for concentrationcalculation. (b) We quantified either the DNA Bit 1 or the denatured DNA Bit 0 (which also exists in single-stranded state in solution) with reporters carrying a pair of fluorophore and quencher. The only difference of these reporters from the reporters used on the paper ticket is: the poly-T tail was removed from U*S3* while a quencher was attached to the 5' end of the U*S3*. When corresponding DNA Bit 1 strand (or denatured DNA Bit 0) is present, it will displace the quencher strand so the fluorophore can light up and give signal. The preparation and annealing protocols of the reporters were identical to the paper ticket reporters. Each DNATag sample was quantified in four wells in the plate reader, two of which were loaded with the S3 reporter, and the other two with S5 reporter. Duplication is for demonstration of robustness. (c) Raw fluorescence signals of all samples. 'PC' stands for positive control, and 'NC' stands for negative control. The reporters were loaded at the start, and the DNATag samples were added after 5 min. (d) Concentrations of the DNA Bit 1 or denatured DNA Bit 0 calculated by linear fitting their induced fluorescence intensities to the positive and negative controls. The 0-day datapoints of Bit 1 were higher because the 0-day DNATags were directly added to the reporter plate after preparation, so they did not go through the drying and retrieval steps and thus did not have any loss from retrieval. According to the measurement, DNATags did not show any sign of degradation or denaturation at dry storage up to 15 days.

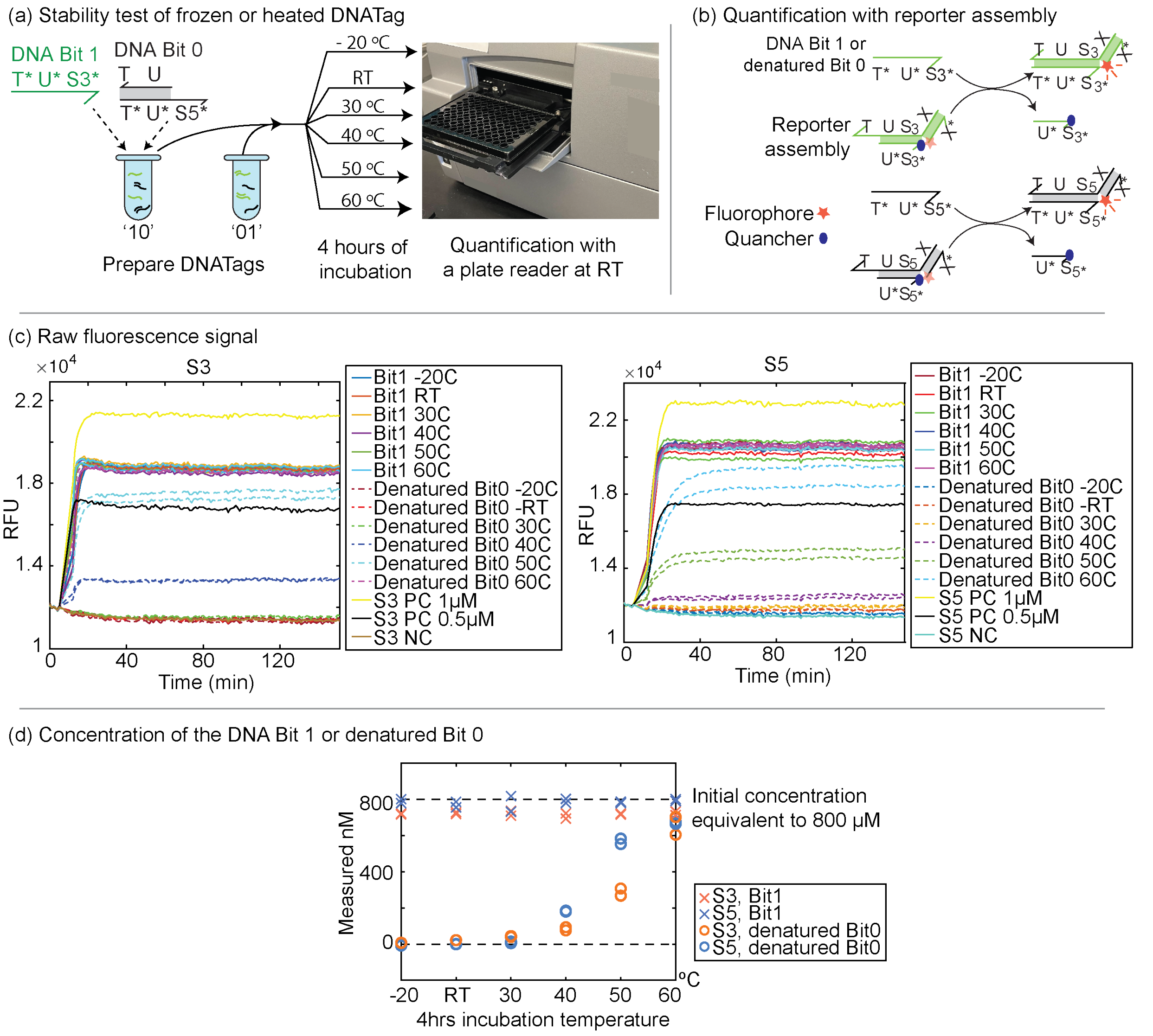

Figure S9: Stability test of DNATags after frozen or heated. (a) Experimental workflow of stability test under varied temperatures. We prepared two 2-bit DNATag solutions '10' and '01' with DNA Bit strands carrying S3* and S5* domains, representing the two digits respectively. The DNATag solutions were freshly prepared and incubated at varied temperatures including -20ºC, room temperature (RT), 30ºC, 40ºC, 50ºC, and 60 ºC for 4 hours. Then, all DNATags were quantified using the corresponding fluorescent reporters in a microplate reader. Positive controls (pure DNA Bit 1 strand at 1μM and 0.5 μM) and negative controls (buffer without any DNA) were also presented for concentration calculation purposes. (b) We quantified either the DNA Bit 1 or the denatured DNA Bit 0 (which also exists in single-stranded state in solution) with reporters carrying a pair of fluorophore and quencher. The only difference of these reporters from the reporters used on the paper ticket is: the poly-T tail was removed from U*S3* while a quencher was attached to the 5' end of the U*S3*. When corresponding DNA Bit 1 strand (or denatured DNA Bit 0) is present, it will displace the quencher strand so the fluorophore can light up and give signal. The preparation and annealing protocols of the reporters were identical to the paper ticket reporters. Each DNATag sample was quantified in four wells in the plate reader, two of which were loaded with the S3 reporter, and the other two with S5 reporter. Duplication is for demonstration of robustness. (c) Raw fluorescence signals of all samples. 'PC' stands for positive control, and 'NC' stands for negative control. The reporters were loaded at the start, and the DNATag samples were added after 6 min. (d) Concentrations of the DNA Bit 1 or denatured DNA Bit 0 calculated by linear fitting their induced fluorescence intensities to the positive and negative controls. According to the result, no degradation or denaturation of DNA Bits occured after frozen. When the temperature exceeded 30ºC, the DNA Bit 0 started to denature and resulted in false positive signal from the reporters. The raised temperature did not affect DNA Bit 1.

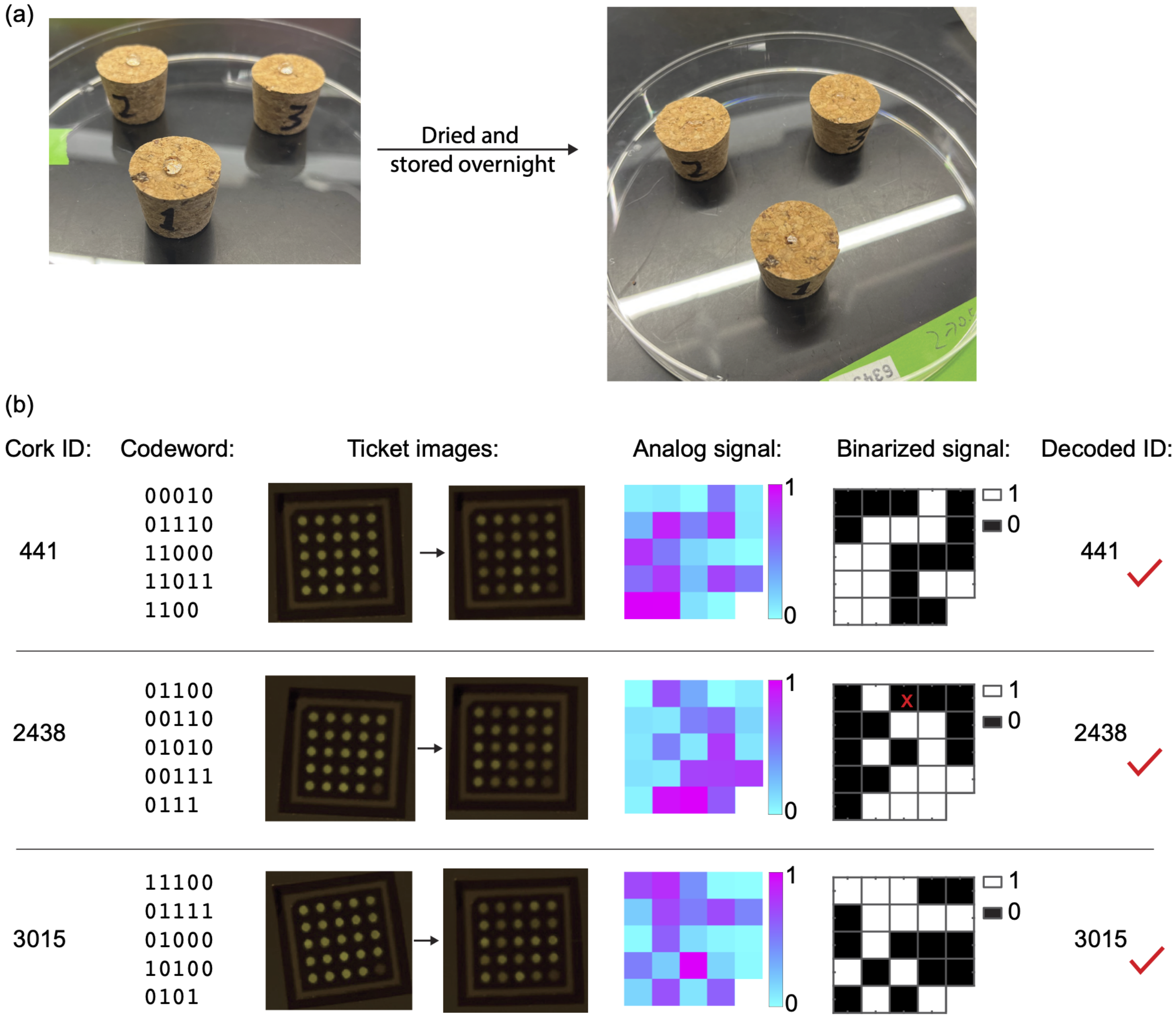

Figure S10: End-to-end workflow labeling corks with randomly generated DNATags. (a) Photos of the corks being labeled with DNATags. Three corks were labeled with three randomly generated DNATags. (b) Readout results from the three labeled corks. All three cork IDs were accurately decoded from the DNATags.

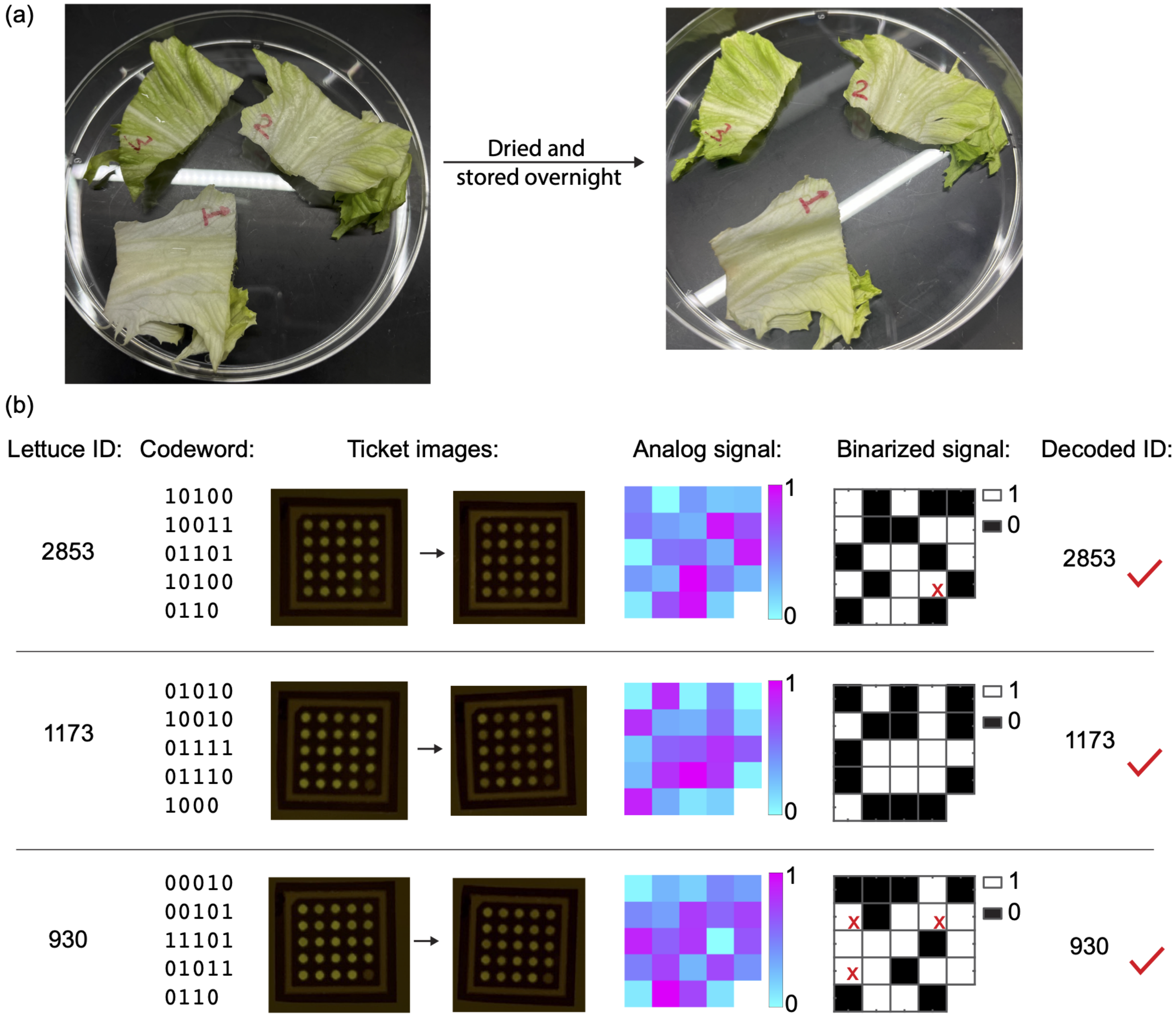

Figure S11: End-to-end workflow labeling lettuce with randomly generated DNATags. (a) Photos of the pieces of lettuce being labeled with DNATags. Three pieces were labeled with three randomly generated DNATags. (b) Readout results from the three labeled corks. All three lettuce IDs were accurately decoded from the DNATags.

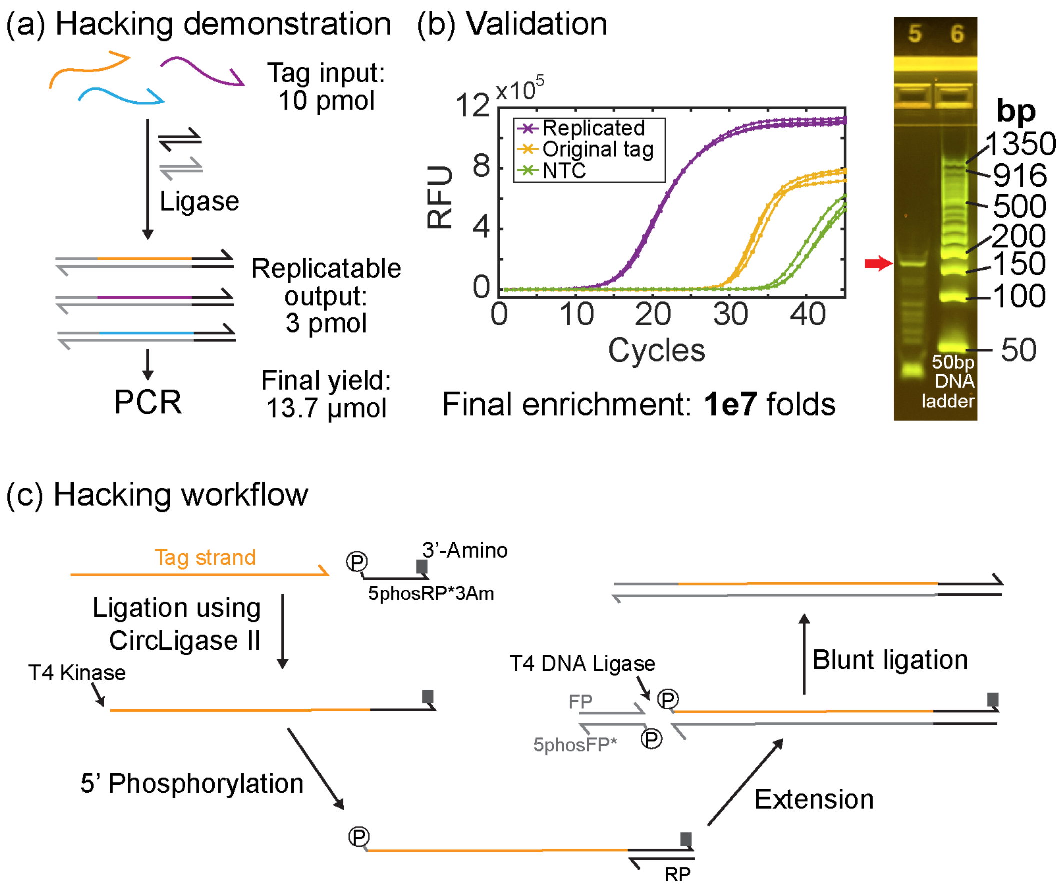

Figure S12: Universal forgery demonstration. (a) Arbitrary ssDNA tags can be easily forged by attaching artificial primer regions to their both ends followed by PCR. (b) The forged replicates were confirmed using the original taggant readout assay and electrophoresis. (c) Detailed experimental workflow of universal forgery. The input DNA tag is first incubated with 5phosRP*3AM and CircLigase II. The product is column purified and then incubated with NEB T4 Kinase. After another column purification, the product is mixed with RP and DNA polymerase and the mixture goes through one PCR cycle for double strand extension. Another column purification is per- formed, and the product is incubated with FP, 5phosFP*, and T4 DNA Ligase. Then after a final column purification, we acquire the forged replicates that are ready for PCR using primers FP and RP.

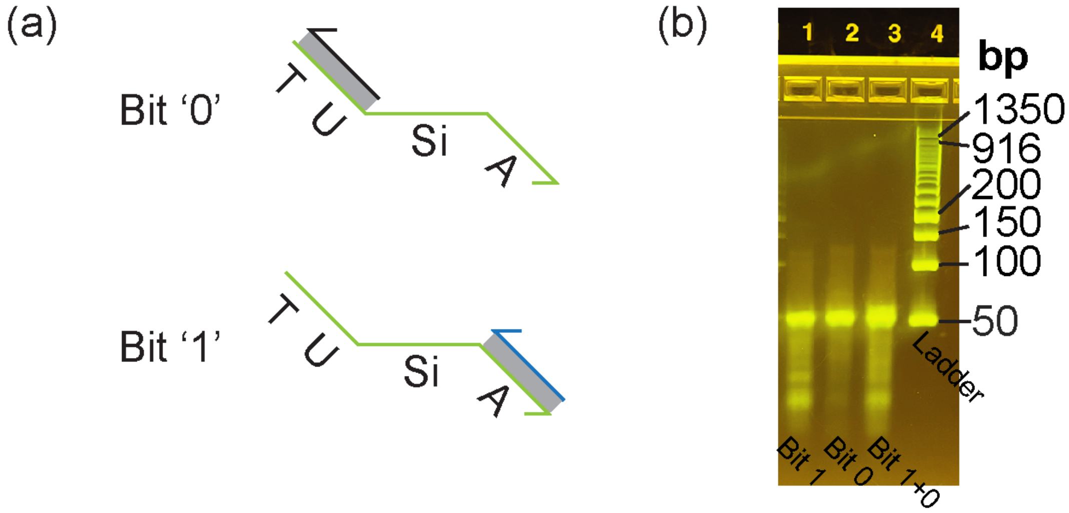

Figure S13: Molecular weight balancing to prevent gel separation of DNAbits 1s and 0s. (a) An auxiliary overhang 'A' could be attached to the 3' end of each DNA bit strand. DNA Bit '1's will have a short 'A*' oligo hybridized to its 'A' tail so the molecular weights of the DNA Bits '0' and '1' are the same. (b) Gel electrophoresis could not distinguish DNA Bits '1' and '0'.

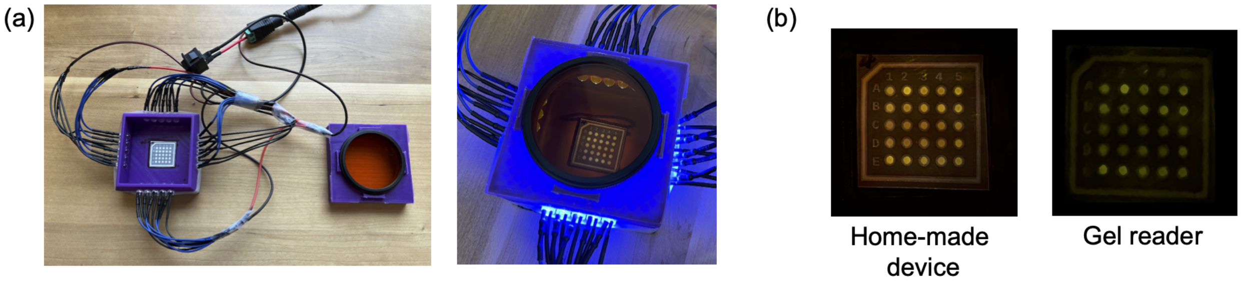

Figure S14: Home-made inexpensive device for capturing fluorescence images. (a) The device consists of 20 blue LEDs (12V input, 5 LEDs on each side of the ticket), one orange filter (Tiffen 49mm 21 Filter purchased from Amazon), one 12V power supply (for powering all 20 LEDs), and some 3D printed frame structure. The total cost of all components is $50. (b) Fluorescence images of the same DNATag reading ticket via the home-made device versus the gel reader.

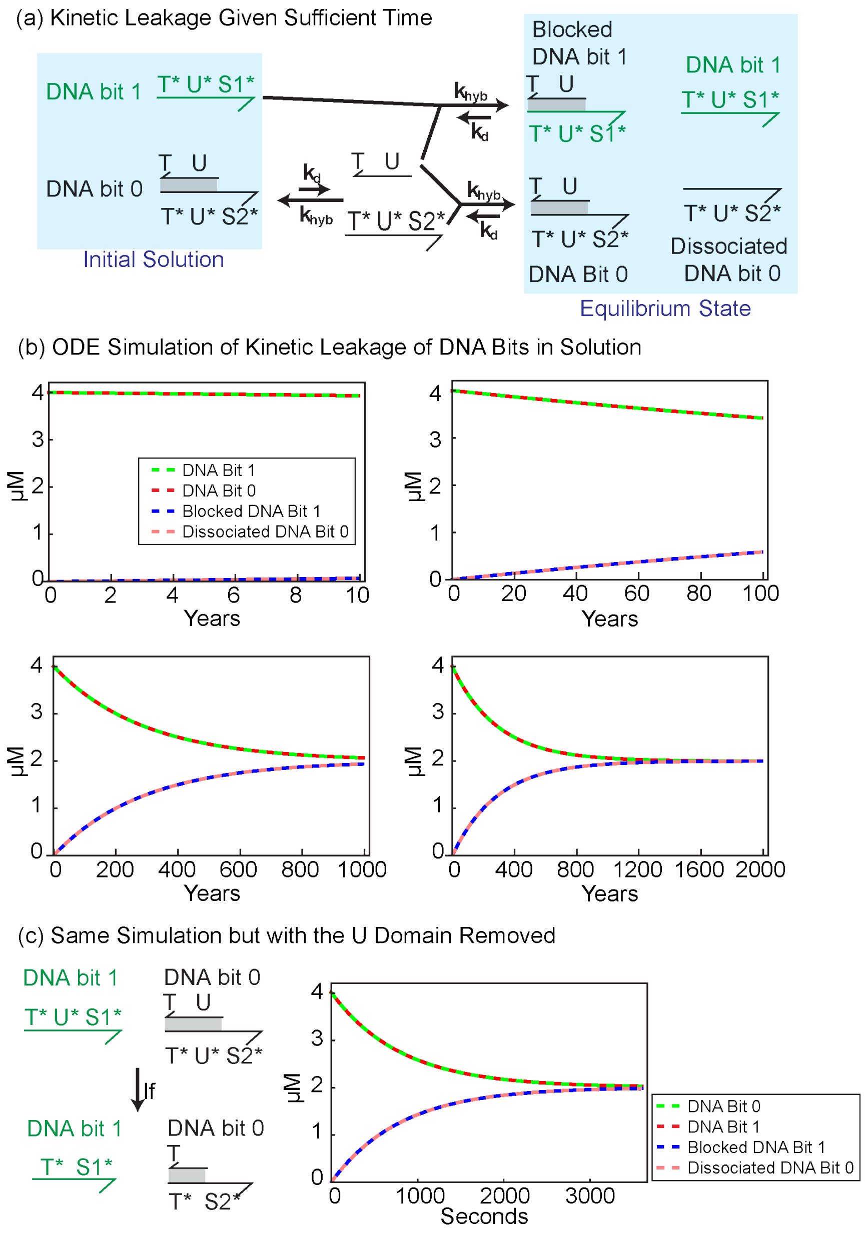

Figure S15: Ordinary differential equation (ODE) simulation of DNA Bits 0 and 1 in solution. (a) The double stranded state of DNA Bits 0 is impermanent: given sufficient time, the blocker strand $< TU >$ will dissociate from DNA Bit 0 and free floating blocker strands will randomly hybridize to the original DNA Bit 1 $< T^*U^*S1^* >$ or the dissociated DNA Bi 0 $< T^*U^*S2^* >$. Thus the solution will relax to the equilibrium state. The unimolecular mechanism is well understood and modeled, and is primarily rate-limited by dissociation constant k_d. The value of k_{hyb} is assumed to be $10^6 M^{-1}s^{-1}$, and the value of k_d is calculated based on $k_d = \frac{k_{hyb}}{e^{-\Delta G^o/R\tau}}$, where $\tau = 273.15 + 23$ is the temperature in Kelvin, R is the ideal gas constant (Boltzmann constant), and ΔG^o is the standard free energy of formation of the duplex formed by the blocker strand $< TU >$ and the binding domain U^*T^* of DNA Bits. The value of ΔG^o is calculated based on the sequence of $< TU >$ and published DNA thermodynamics parameters. (b) The ODE simulation was carried out in Mathematica using scripts developed by Soloveichik et al. [1]. According to the simulation results, the kinetic leakage is barely noticeable in 10 years. (c) ODE simulation result if we remove the U domain. The solution reaches the equilibrium in just 1 hour. This proved that the U domain helped stabilize the DNA Bits.

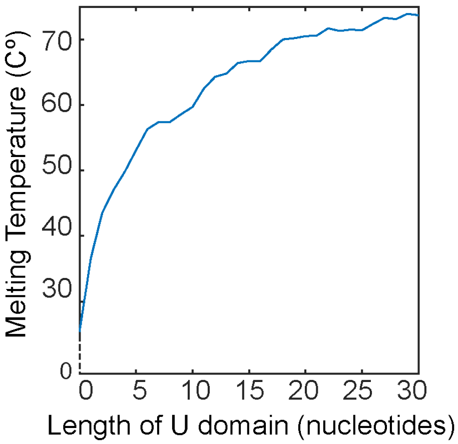

Figure S16: Calculated melting temperature of the double-stranded domain on DNA Bit 0 with varied lenghts of U^* domain.

Supplementary References

[1] David Soloveichik, Georg Seelig, and Erik Winfree. Dna as a universal substrate for chemical kinetics. *Proceedings of the National Academy of Sciences*, 107(12):5393–5398, 2010.
