# Supplementary material for: Hybridization-encoded DNA tags with paper-based readout for anti-forgery raw material tracking
Source: Nat Commun. 2025 Jul 1;16:5832. doi: 10.1038/s41467-025-60282-7 (PMC12214956; doi:10.1038/s41467-025-60282-7)
Supplement: Supplementary file 2 — Reporting Summary [file 41467_2025_60282_MOESM2_ESM.pdf]

nature portfolio

Corresponding author(s): Yuan-Jyu Chen

Last updated by author(s): Mar 28, 2025

Reporting Summary

Nature Portfolio wishes to improve the reproducibility of the work that we publish. This form provides structure for consistency and transparency in reporting. For further information on Nature Portfolio policies, see our Editorial Policies and the Editorial Policy Checklist.

Statistics

For all statistical analyses, confirm that the following items are present in the figure legend, table legend, main text, or Methods section.

n/a	Confirmed	
☐	☒	The exact sample size (*n*) for each experimental group/condition, given as a discrete number and unit of measurement
☐	☒	A statement on whether measurements were taken from distinct samples or whether the same sample was measured repeatedly
☐	☒	The statistical test(s) used AND whether they are one- or two-sided<br>*Only common tests should be described solely by name; describe more complex techniques in the Methods section.*
☐	☒	A description of all covariates tested
☐	☒	A description of any assumptions or corrections, such as tests of normality and adjustment for multiple comparisons
☐	☒	A full description of the statistical parameters including central tendency (e.g. means) or other basic estimates (e.g. regression coefficient) AND variation (e.g. standard deviation) or associated estimates of uncertainty (e.g. confidence intervals)
☐	☒	For null hypothesis testing, the test statistic (e.g. *F*, *t*, *r*) with confidence intervals, effect sizes, degrees of freedom and *P* value noted<br>*Give P values as exact values whenever suitable.*
☒	☐	For Bayesian analysis, information on the choice of priors and Markov chain Monte Carlo settings
☒	☐	For hierarchical and complex designs, identification of the appropriate level for tests and full reporting of outcomes
☒	☐	Estimates of effect sizes (e.g. Cohen's *d*, Pearson's *r*), indicating how they were calculated

Our web collection on statistics for biologists contains articles on many of the points above.

Software and code

Policy information about availability of computer code

Data collection	Data collection procedure and related devices and software are described in detail in the 'Method' section of the manuscript.
Data analysis	Custom code run on MATLAB R2021a was used for data analysis.<br>Custom code run on Jupyter Notebook was used for data analysis.<br>Software ImageJ (version 1.50d) was used for data analysis.<br>All custom code scripts are available at GitHub ath URL: https://github.com/microsoft/DNATagging/.<br>All drawings, diagrams and flowcharts were created using Adobe Illustrator.

For manuscripts utilizing custom algorithms or software that are central to the research but not yet described in published literature, software must be made available to editors and reviewers. We strongly encourage code deposition in a community repository (e.g. GitHub). See the Nature Portfolio guidelines for submitting code & software for further information.

Data

Policy information about availability of data

All manuscripts must include a data availability statement. This statement should provide the following information, where applicable:

- Accession codes, unique identifiers, or web links for publicly available datasets
- A description of any restrictions on data availability
- For clinical datasets or third party data, please ensure that the statement adheres to our policy

All data that supports the findings of this study is available on GitHub at the following URL: https://github.com/microsoft/DNATagging/ or on Zenodo via DOI: https://doi.org/10.5281/zenodo.14933948.

Research involving human participants, their data, or biological material

Policy information about studies with human participants or human data. See also policy information about sex, gender (identity/presentation), and sexual orientation and race, ethnicity and racism.

Reporting on sex and gender	n/a
Reporting on race, ethnicity, or other socially relevant groupings	n/a
Population characteristics	n/a
Recruitment	n/a
Ethics oversight	n/a

Note that full information on the approval of the study protocol must also be provided in the manuscript.

Field-specific reporting

Please select the one below that is the best fit for your research. If you are not sure, read the appropriate sections before making your selection.

☒ Life sciences ☐ Behavioural & social sciences ☐ Ecological, evolutionary & environmental sciences

For a reference copy of the document with all sections, see nature.com/documents/nr-reporting-summary-flat.pdf

Life sciences study design

All studies must disclose on these points even when the disclosure is negative.

Sample size	The 5-by-5 array size of the DNATag reading ticket was determined because it was sufficient for encoding typical barcodes. The sample size of the multi-bit experiments was selected to provide sufficient statistical significance.
Data exclusions	No data were excluded from analysis.
Replication	We ran the specificity test in duplicates and the kinetics test three times with varied DNA Bits. We ran multi-bit DNATag reading experiments for at least three times per controlled experimental setting. We duplicated all data reading in the microplate reader experiments. All attempts at replication were successful.
Randomization	All multi-bit DNATag were encoded with randomly generated datawords.
Blinding	We did not involve blinding because it was not necessary. All datawords were randomly generated in silico. The DNATag is a solution of mixed DNA Bits and any mis-handling could only result in false negative results. We acquired most of our conclusions based on positive results, and we excessively conducts replication and randomization to ensure reliability.

Reporting for specific materials, systems and methods

We require information from authors about some types of materials, experimental systems and methods used in many studies. Here, indicate whether each material, system or method listed is relevant to your study. If you are not sure if a list item applies to your research, read the appropriate section before selecting a response.

Materials & experimental systems

n/a	Involved in the study
[x]	[ ] Antibodies
[x]	[ ] Eukaryotic cell lines
[x]	[ ] Palaeontology and archaeology
[x]	[ ] Animals and other organisms
[x]	[ ] Clinical data
[x]	[ ] Dual use research of concern
[x]	[ ] Plants

Methods

n/a	Involved in the study
[x]	[ ] ChIP-seq
[x]	[ ] Flow cytometry
[x]	[ ] MRI-based neuroimaging

Plants

Seed stocks	Lettuce pieces were used as a substrate for DNATag in our experiment. The lettuce was purchased from a local QFC grocery store.
Novel plant genotypes	n/a
Authentication	n/a
